# Under the hood of statistical learning: A statistical MMN reflects the magnitude of transitional probabilities in auditory sequences

**DOI:** 10.1038/srep19741

**Published:** 2016-02-02

**Authors:** Stefan Koelsch, Tobias Busch, Sebastian Jentschke, Martin Rohrmeier

**Affiliations:** 1 University of Bergen, Department for Biological and Medical Psychology, Bergen, 5009, Norway; 2 Freie Universität Berlin, Department for Educational Sciences and Psychology, Berlin, 14195, Germany; 3 Technische Universität Dresden, Institut für Kunst- und Musikwissenschaft, Dresden, 01219, Germany

## Abstract

Within the framework of statistical learning, many behavioural studies investigated the processing of unpredicted events. However, surprisingly few neurophysiological studies are available on this topic, and no statistical learning experiment has investigated electroencephalographic (EEG) correlates of processing events with different transition probabilities. We carried out an EEG study with a novel variant of the established statistical learning paradigm. Timbres were presented in isochronous sequences of triplets. The first two sounds of all triplets were equiprobable, while the third sound occurred with either low (10%), intermediate (30%), or high (60%) probability. Thus, the occurrence probability of the third item of each triplet (given the first two items) was varied. Compared to high-probability triplet endings, endings with low and intermediate probability elicited an early anterior negativity that had an onset around 100 ms and was maximal at around 180 ms. This effect was larger for events with low than for events with intermediate probability. Our results reveal that, when predictions are based on statistical learning, events that do not match a prediction evoke an early anterior negativity, with the amplitude of this mismatch response being inversely related to the probability of such events. Thus, we report a statistical mismatch negativity (sMMN) that reflects statistical learning of transitional probability distributions that go beyond auditory sensory memory capabilities.

Humans are sensitive to regularities within their environment and pick up statistical structures without explicit intent. This ability is explored within the frameworks of statistical learning^1^ and implicit learning^2^, both of which have been argued to investigate the same underlying learning phenomenon^3^,^4^. Although statistical learning appears to be domain-general^5^, it has most prominently been investigated in the context of language acquisition, especially word learning (for a review see ref. 6), as well as music^7^,^8^,^9^,^10^,^11^ (for reviews see refs 12, 13, 14). With regard to statistical learning paradigms, word learning has been argued to be grounded, at least in part, in sequence prediction: In a continuous stream of syllables, sequences of events linked with high statistical conditional probability likely correspond to words, whereas syllable transitions with low predictability may likely be indicative of word-boundaries^14^,^15^,^16^. Thus, tracking conditional probability relations between syllables has been regarded as highly relevant for the extraction of candidate word forms^17^,^18^.

In addition, integrating information across the extracted units eventually reveals distributional properties^19^,^20^. Extracted statistical properties provide an important basis for predictions which guide the processing of sensory information^20^,^21^,^22^. Stimuli that are hard to predict (e.g. the syllable after a word boundary) have been hypothesized to increase processing load^21^,^22^. Such an increase in processing load has been found to be reflected neurophysiologically in event-related brain potential (ERP) components such as the N100 and the N400: During successful stream segmentation, word-onsets evoke larger N100 and N400 ERPs compared to more predictable positions within the word in adults (e.g. refs 14,23, 24, 25, 26, 27), and similar ERP responses have been observed even in newborns^28^.

Although the statistical learning paradigm has been extensively used (typically using sequences of triplet stimuli), only few neurophysiological studies have varied the occurrence probability of single events within triplets^29^,^30^,^31^,^32^. A magnetoencephalograpic (MEG) study by Furl *et al*.^31^ used second-order Markov chains in which any pair of tones predicted the next tone with 90% probability. The remaining 10% of tones were, thus, unexpected (given successful learning of the second-order statistics). Such unexpected events elicited stronger magnetic fields than expected events in the time range between ~150–250 ms. An MEG study by Paraskevopoulos *et al*. used sequences of tone triplets, with triplet endings occurring with either high or low probability (thus, low and high probability triplets differed only with regard to the last tone of a triplet)^32^. Final tones with low probability elicited an enhanced P50m and a negativity around 150 ms (the latter was referred to by the authors as mismatch negativity, MMN). Finally, two MEG studies by Daikoku *et al*.^31^,^32^ used sequences in which sound pairs predicted a subsequent sound either with high (80%) or low probability (second-order Markov chains, similar to the study by Furl *et al*.)^31^. One study used sounds with different pitches^29^, and another study also used sounds with different phonemes, as well as pitches and phonemes combined^30^. In both studies, low-probability sounds elicited a stronger MEG response than high-probability sounds in the time-window from 100–220 ms, reminiscent of an MMN. Remarkably, these four MEG studies (refs 29, 30, 31, 32) are—to the best of our knowledge—the only studies reporting brain responses to low-probability items occurring instead of high-probability items within a statistical learning paradigm. This scarcity of studies is surprising, because processing of unexpected events, i.e. of events violating predictions based on prior (implicit) learning of statistical regularities, is a fundamental aspect of our everyday life.

Beyond the statistical learning paradigm, multiple types of predictive processes have been investigated, and several types of early mismatch responses have been reported in response to unpredicted events: For example, violations of statistical regularities that are established on-line, on a moment-to-moment basis, elicit a mismatch negativity (MMN), and the regularities underlying the generation of the physical MMN^33^ and the abstract-feature MMN^34^ are thought to be represented mainly in sensory memory^35^,^36^,^37^,^38^. If local transition probabilities stored in auditory sensory memory change, the sensory representations of the new transition probabilities (and even of auditory feature contingencies) are dynamically updated, as reflected in MMN responses in the time range between ~100–200 ms^39^,^40^. By contrast, deviants in statistical learning paradigms, like those employed in the MEG studies described above^29^,^30^,^31^,^32^, require an extended period of learning, and the mismatch response associated with statistical learning reflects the processing of local dependencies based on (implicit) knowledge about statistical regularities. That is, the mismatch response associated with statistical learning is based on memory representations beyond the capabilities of sensory memory.

Other early mismatch responses observed beyond statistical learning paradigms are (early) left anterior negativities^41^, the syntactic MMN^42^,^43^, and the early right anterior negativity (ERAN)^44^. The (early) left anterior negativities and the syntactic MMN have been reported in language-experiments, and are taken to reflect syntactic processing including the processing of irregular local dependencies (e.g. morpho-syntactic irregularities such as “he come” and “we comes”). However, these ERP components have not been investigated in statistical learning experiments, and they most probably do not only reflect processing of local dependencies, but also processing of syntactic (e.g. morpho-syntactic) information. The ERAN is evoked by music-syntactically irregular events within chord sequences or melodies. However, previous ERAN studies do not inform us about the processing of local dependencies comparable to those used in statistical learning paradigms, because in all previous ERAN studies using local irregularities, local irregularity confounded with hierarchically organized non-local dependencies^45^. For example, in a normal five-chord-cadence starting and ending on a tonic (as, e.g., in ref. 46), the final tonic following the penultimate dominant also hierarchically prolongs the initial tonic. Therefore, a final chord other than the tonic is not only locally less regular, but also violates the hierarchical organization of the harmonic sequence established by the first four chords.

To investigate ERP correlates of processing events that occur instead of predicted high-probability events within a statistical learning paradigm, we constructed auditory sequences using triplets of sounds. The triplets exhibited three different degrees of internal transition probability (see Fig. 1). They were randomly concatenated in a continuous stream, and had endings with high, intermediate as well as low probability (see Fig. 1). Participants passively listened to this stream, and our aim was to investigate the electrical brain responses evoked by the triplet endings (that is, ERPs evoked by triplet endings with high, intermediate, and low probability). Thus, we employed a novel statistical oddball paradigm that advances the traditional statistical learning paradigm and the methods used in previous neurophysiological studies. Note that, similar to the above-mentioned MEG studies using statistical learning paradigms^29^,^30^,^31^,^32^, we were primarily interested in investigating brain responses to low probability items at a position with relatively low entropy, rather than triplet segmentation processes (used as a paradigm to investigate learning processes associated with word segmentation)^14^,^23^,^24^,^25^,^26^.

We used synthetic timbres as stimuli, instead of phonemes or pitches, because both Western tonal music and language exhibit a strong hierarchical organization^47^,^48^,^49^. Thus, it can be guaranteed that our stimuli exclude potential interference by long-term representations of hierarchically organized regularities (due to the lack of a pre-existing hierarchically organized rule system for our synthetic timbres).

We hypothesized that participants would incidentally establish a model of the transitional probability distribution over triplet endings. Because model predictions guide subsequent processing, endings with lower probability should be less expected, and the effort to process items should be inversely related to the magnitude of their probability^50^. Using ERPs, we investigated the neurophysiological correlates of different magnitudes of probability (and thus of differences in processing load). Specifically, based on the results of the MEG studies on statistical learning described above^29^,^30^,^31^,^32^, we expected an early negativity in reaction to low probability triplet endings. In addition, we tested whether this negativity would have a larger amplitude when elicited by low compared to intermediate probability items. Thus, results were supposed to inform us about neurophysiological correlates of the processing of local dependencies, using a novel statistical learning paradigm and a hierarchy-free stimulus environment.

## Materials and Methods

### Participants

18 adults (9 females; mean age = 25.6 years, *SD* = 4.3) were recruited at the Freie Universität Berlin. All participants were right-handed (mean laterality quotient = 95.2, 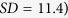 according to the Edinburgh Handedness Inventory^51^. None of the participants had hearing impairment, absolute pitch, history of neurological disease, or musical training besides regular school lessons (according to self-report).

### Ethics Statement

The study was carried out in accordance with the guidelines of the Declaration of Helsinki, and approved by the ethics committee of the Department of Educational Sciences and Psychology of the Freie Universität Berlin. Participants provided written informed consent before the experiment.

### Stimuli

#### Timbre Triplets

**Figure 1:**
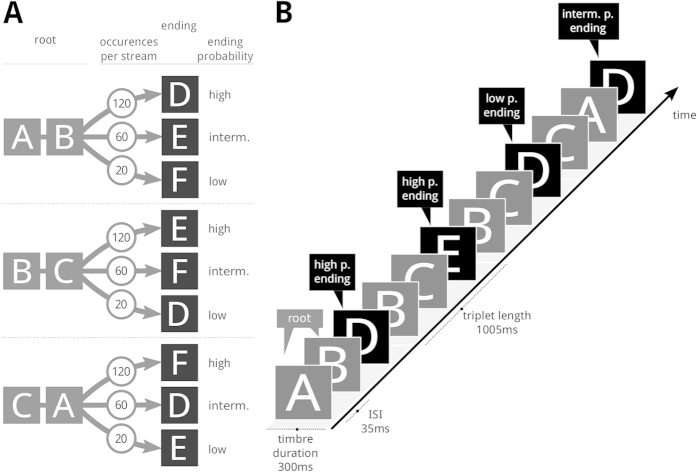
Arrangement of timbres into triplets and streams. (**A**) Each of three triplet roots (AB, BC, CA) consisted of two timbres. Three different timbres were attached to each root (triplet endings: D, E, F), leading to nine timbre triplets. Across the exposition phase (duration ~1 h), triplets were presented with different frequencies of occurrence in a way that each root would be followed by a specific triplet ending with either high, intermediate, or low probability (numbers in circles indicate total number of presentations of a triplet during the exposition phase). (**B**) Timbre triplets were concatenated into a pause-free, pseudorandom stream. ERPs were calculated for triplet endings (i.e., for the final item of each triplet, indicated by the black squares), and for triplet roots (i.e., for the first two items of each triplet, indicated by the gray squares). Letters A to F refer to the six timbres used in this study; interm.: intermediate; p.: probability).

Six timbre sounds were selected from a study by McAdams *et al*. (ref. 52). All sounds were equalized in pitch and loudness. Each sound had a duration of 300 ms, including a fadeout of 15 ms. Each timbre was followed by a 35 ms pause. The six timbres were reminiscent of Bassoon, Clarinet, Harpsichord, Trombone, Harp, and Striano (bowed string/piano). We will refer to them with letters A to F (see also Fig. 1A).

The six timbre sounds (A to F) were combined into sound triplets (Fig. 1A), as decribed in the following. Timbres A, B, and C were used for the first two timbres of each triplet: Each of these three timbres (A, B, C) appeared once in the first and once in the second position (AB, BC, and CA, see also Fig. 1A). In the following, we will refer to the first two sounds of a triplet as “*root*”. Thus, three timbres (A, B, C) were used to create three roots (AB, BC, CA). The remaining timbres (D, E, F) served as triplet endings for each of the three roots, resulting in nine unique triplets (see also Fig. 1A).

#### Triplet Sets

The nine triplets were divided into three sets of triplets: one set with low frequency of occurrence (10%), one with intermediate frequency of occurrence (30%), and one with high frequency of occurrence (60%). E.g., the set with high frequency of occurrence comprised triplets ABD, BCE, and CAF (see also Fig. 1A). Note that each set contained each root and each ending exactly once. Thus, constructing the triplet sets did not affect the presentation frequencies of individual timbres, roots or endings, as these were evenly distributed across sets. In other words, the transitional probability of a triplet ending, given the first two timbres of a triplet, depended on which of the three sets this specific triplet was in. Accordingly, we will refer to sets and triplet endings as low, intermediate, and high probability sets and endings.

#### Triplet Streams

The triplets were concatenated to construct five pause-free timbre streams. Note that the interstimulus interval between two successive timbres was always 35 ms. Each stream consisted of 600 triplets and was 10 min long. Within each stream, triplets from the low, intermediate, and high probability sets were presented with a ratio of 1:3:6. Concretely, each triplet was presented 20, 60 or 120 times, respectively (see Fig. 1B). The sequence of triplets within each stream was pseudorandomized: Triplets were never repeated directly, and triplets from the low probability set were separated by at least three triplets from another set. Four additional timbres were used to create two completely random streams used only for demonstration and practice. They were edited like the six timbres used in the reqular streams. Each practice stream consisted of 90 sounds and was 30 s long.

### Procedure

Participants sat in a sound attenuated room. An experimenter was present at all times. Participants were not informed about the intent of the study, nor the regularities within the stimuli. Sounds were presented via headphones at a normal comfortable intensity level (around 50 dB SPL). The experiment consisted of two parts, an exposition phase (~1 h) during which the electroencephalogram (EEG) was recorded, and a behavioral task (~8 min).

#### Exposition Phase

The first part of the experiment was the exposition phase. The five timbre streams were presented as blocks, thus there were five blocks of stimuli (with a duration of 50 min in total). Between blocks there were pauses, and the length of each pause was determined by the participant. During listening, participants watched a muted nature film (“Winged Migration”, Sony Pictures Classics, 2003), presented at ~60 cm distance within a horizontal visual angle of ~17°.

As a cover task, and to control that participants processed the stimulus, 60 of the 9000 timbres (0.67%) were presented to one ear only. Participants were instructed to quickly press the space key in response to such acoustical deviants. The side of presentation was randomized. The acoustical deviants were only used for the cover task, and excluded from the ERP data analysis. Before the exposition phase, the two practice streams were presented to familiarize participants with this task. Each practice stream contained three acoustical deviants.

#### Goodness-of-fit and Confidence Ratings

After the exposition phase (which lasted ~1 h), a behavioral test assessed the ability to differentiate triplet endings based on their frequency of occurrence with which they were presented during the exposition phase. Participants were presented with 36 trials. In each trial, participants were presented with two triplets (e.g. ABD and ABE). The first two sounds of these two triplets were the same (i.e., both triplets had the same root, e.g. AB), thus only the third sound (i.e., the triplet ending) differed between the two triplets. There was a 335 ms pause between the two triplets. Participants were asked which triplet-ending they “found to fit better” (two-alternative, forced-choice goodness-of-fit rating), and how confident they were with regard to their choice. The confidence ratings were obtained to assess whether participants were aware of any knowledge guiding their choices. Confidence ratings were obtained using a six-point scale, ranging from “guessing” to “absolutely certain”. The ending that had occurred more often during the exposition phase (see above) was defined as better fitting, and thus as correct. Participants were rewarded with 10 cents for each correct choice. Feedback about the winnings was given after the last trial only. For each of the three roots (AB, BC, CA, see also Fig. 1A), three such comparisons were possible (high vs. intermediate, high vs. low., and intermediate vs. low probability endings). All of these nine comparisons of triplet endings were presented four times, in random order. Comparisons involving the same root were not repeated in consecutive trials and the order of presentation of the endings was counterbalanced.

### Data Recording and Analysis

#### EEG recording

EEGs were recorded from 59 electrodes, cap-mounted in compliance with the extended 10-10 system (Fpz, Fp1, Fp2, AF3, AF4, AF7, AF8, Fz, F1, F2, F3, F4, F5, F6, F7, F8, FC1, FC2, FC3, FC4, FC5, FC6, FT7, FT8, Cz, C1, C2, C3, C4, C5, C6, T7, T8, CPz, CP1, CP2, CP3, CP4, CP5, CP6, TP7, TP8, Pz, P1, P2, P3, P4, P5, P6, P7, P8, POz, PO3, PO4, PO7, PO8, Oz, O1, O2), with additional electrodes placed on the right and left mastoids, as well as on the neck. The left mastoid electrode was used as reference, and the neck electrode was used as ground. Horizontal and vertical electro-occulograms (EOGs) were recorded bipolarly with electrodes placed at the outer canthi of the eyes, and above and below the right eye. Electrode impedance was kept below 5 *k*Ω. Signals were recorded using Brain Products Vision Recorder (Brain Products GmbH, Munich, Germany) with a 0.1 Hz to 1000 Hz bandpass filter and 500 Hz sampling rate.

#### EEG Data Analysis

EEG-data were analyzed using EEGLAB^53^. After data acquisition, EEG-data were re-referenced to the algebraic mean of the left and right mastoid electrodes and filtered with a 0.5 Hz high-pass filter (550 point, finite impulse response, Blackman) and a 30 Hz low-pass filter (2750 points, finite impulse response, Blackman). Epochs containing acoustical deviants or button presses were excluded from further analysis. Data containing artifacts were rejected by removing sampling points whenever the standard deviation within a 200 or 800 ms gliding window exceeded 25 *μV* at any electrode channel (including the EOG channels). Artifact rejections were visually controlled by the second author. Furthermore, Independent Component Analysis was used to remove eye and muscle artifacts. ERPs were calculated for low, intermediate, and high probability triplet endings. In addition, to determine whether the triplet structure of the stimulus stream was represented in participants’ brain responses, ERPs were also calculated for triplet roots (i.e., the first two triplet items) for each block. All ERPs were computed using a 100 ms pre-stimulus baseline.

For statistical evaluation, electrodes were clustered into two regions of interest (ROIs), namely left anterior (FP1, AF3, AF7, F1, F3, F5, FC1, FC3, FC5) and right anterior (FP2, AF4, AF8, F2, F4, F6, FC2, FC4, FC6). The time windows used for statistical analyses (see below) were based on previous results (refs 29, 30, 31, 32, see Introduction) and visual inspection. Using SPSS 23.0 (IBM Corp., Armonk, NY, USA), ERPs elicited by triplet endings were statistically analyzed with a three-way repeated measures multivariate Analysis of Variance (MANOVA) with factors probability (low, intermediate, and high probability ending), block (1 to 5), and hemisphere (left and right ROI) as within-subjects factors. Time windows were 70–130 ms and 130–220 ms. Significant effects of probability were investigated further using user-defined contrasts. ERPs elicited by triplet roots were statistically analyzed with a three-way repeated measures multivariate Analysis of Variance (MANOVA) with factors item position (first and second triplet item), block (1 to 5), and hemisphere (left and right ROI) as within-subjects factors. Time windows were 70–130 ms and 170 to 230 ms.

#### Behavioral Data Analysis

For the evaluation of the cover task (during the exposition phase), button presses within up to three sounds (i.e., 1.34 s) after acoustical deviants were regarded as hits. Choices in the goodness-of-fit ratings (after the exposition phase) were classified as correct if the chosen (“preferred”) triplet ending had higher probability than the rejected one. Otherwise choices were classified as incorrect. Mean correctness was calculated for each participant as the proportion of correct responses to the overall number of responses. Mean correctness was tested against chance level (two-tailed *t*-test, 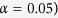. Correctness was also entered into an ANOVA with trial type (trials contrasting either high vs. low, high vs. intermediate, or low vs. intermediate endings) and repetition (four repetitions of nine comparisons of triplet endings) as within-subject factors. Factor repetition was included to guarantee that the frequencies of occurrence during the behavioral test did not modify the learned frequency distributions. In addition, Pearson’s product-moment correlation between goodness-of-fit and confidence ratings was calculated and tested for significance with a two-tailed *t*-test 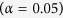 for all trials and for each trial type separately. This analysis was carried out to determine whether participants with higher correctness rates would also be more confident in their judgements. Statistical evaluations of behavioral data were computed using *R*^54^.

## Results

### Acoustical Deviance Detection (Cover Task)

Participants discovered on average 98.9% of the 60 acoustical deviants in the cover task (*SD* = 1.3). No participant had more than two false alarms (*M* = 0.9, *SD* = 0.8). This indicates that participants perceived and responded to the acoustical stimuli despite watching the (muted) movie.

### ERPs of Triplet Endings

**Figure 2:**
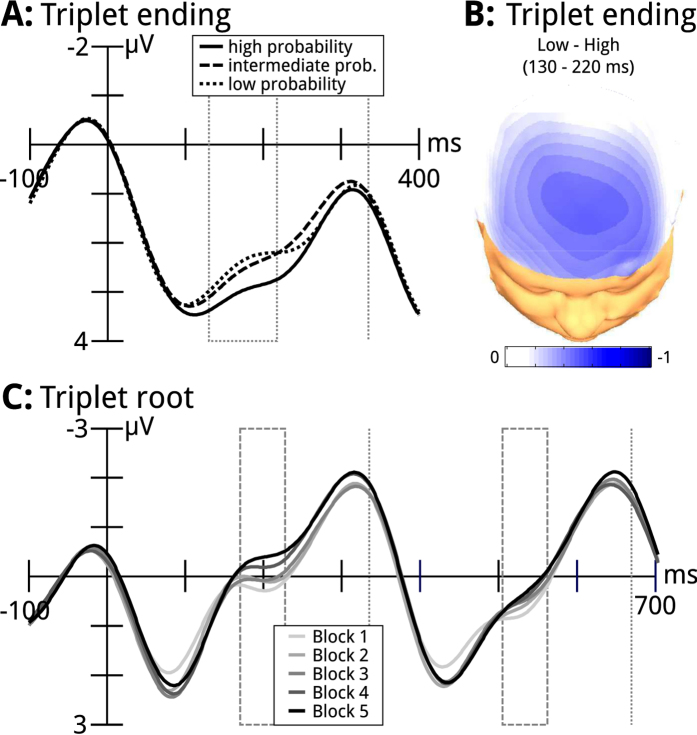
ERP results. (**A**) Mean ERP waves of triplet endings (averaged across participants), recorded at Fz (−100–400 ms relative to the onset of the third triplet item). ERPs are shown separately for high (solid line), intermediate (dashed line), and low probability endings (dottes line). The area within the dotted rectangle indicates the time window used for statistical analysis (130–220 ms). The dotted vertical line indicates the onset of the subsequent timbre (i.e., the beginning of the next triplet). Compared to high probability triplet endings, endings with low and intermediate probability elicited a frontal negativity (this effect was larger for low than for intermediate probability endings). (**B**) Isopotential map showing the scalp distribution of the negativity evoked by low probability endings compared to high probability endings for the time window used for statistical analysis (difference potential: high subtracted from low probability endings). (**C**) Mean ERP waves of triplet roots (i.e., first and second items of triplets), recorded at Fz (−100–700 ms relative to the onset of the triplet root), separately for each of the five blocks. The dashed rectangle indicates the time window used for statistical analysis (170–230 ms). The dashed vertical lines indicate onsets of stimuli (i.e., onset of the second and third triplet item).

Figure 2A shows that the triplet endings elicited a positive-going wave (P1) with a latency of ~100 ms, followed by a negative-going wave that was maximal at ~180 ms over frontal electrodes. The amplitude of this negativity varied with ending probability (see also dotted rectangle in Fig. 2A), being more negative for low than medium probability items, and more negative for medium than high probability items (see also Fig. 2B and Table 1).

**Figure 3:**
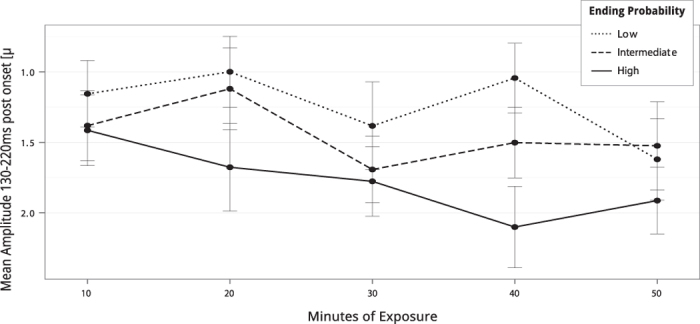
Mean amplitudes 130–220 ms elicited by triplet endings with different probabilities over the course of the experiment. The mean amplitude changed over blocks, following a linear positive trend. Error bars indicate standard error. The amplitude scale is inverted to facilitate comparability with Fig. 2.

A MANOVA with factors probability (low, intermediate, and high probability ending), block (1 to 5), and hemisphere (left and right ROI) for the time window from 130 to 220 ms after the onset of triplet endings showed a significant main effect of probability on mean ERP amplitudes 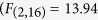, 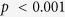, 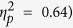. Post-hoc tests using polynomial orthogonal contrasts revealed a significant linear trend 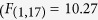, 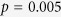, 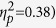. This reflects an amplitude increase of the observed negativity from low over intermediate to high probability endings. Planned comparisons with user-defined contrasts confirmed this increase, revealing that the negativity differed significantly (and with large effect sizes) between low and high probability endings, and between intermediate and high probability endings 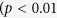 in each test, see Table 2 for details). The difference between low and intermediate probability endings was statistically not significant. Another main effect was observed for the factor block 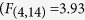, 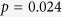, 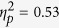; see Fig. 3). Polynomial contrasts revealed a linear trend 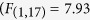, 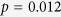, 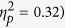. The interaction of the factors probability and block approached statistical significance 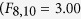, 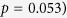.

A MANOVA for the P1 effect with factors probability (low, intermediate, and high probability ending), block (1 to 5), and hemisphere (left and right ROI) for the time window from 70 to 130 ms after the onset of triplet endings did not show a clear effect of probability on mean ERP amplitudes 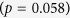, nor any interaction involving item probability.

### ERPs of Triplet Roots

To determine whether the triplet structure of the stimulus stream was represented in participants’ brain responses, we also inspected the ERPs elicited by the first two triplet items (i.e., by the triplet roots, see also gray squares in Fig. 1B). Recall that the first two items were always a subset of the three timbres that could occur at positions one and two of each triplet (Fig. 1A). Therefore, any difference in brain responses between the first two triplet items could only be due to the fact that participants had a cognitive representation of the triplet structure of the stimulus stream. Figure 2C shows that both first and second triplet items elicited a positive-going wave (P1) at ~80 ms after stimulus onset, followed by an N1-like wave (indicated by the dashed rectangles) at ~200 ms, and another (N2-like) negative wave peaking at ~310 ms. The N1-like wave clearly differed between the first and the second triplet items (whereas P1 and N2-like waves were similar for both triplet items). In addition, the N1-like wave increased systematically across blocks when elicited by the first, but not by the second item, probably reflecting developing word segmentation across blocks.

A MANOVA with factors item position (first and second triplet item), block (1 to 5), and hemisphere (left and right ROI) for the time window from 170 to 230 ms after the onset of the first and second triplet items showed significant effects of item position (reflecting that the amplitude of the N1-like wave differed between first and second triplet item, 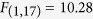, 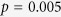, 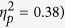, block (reflecting that the amplitude of the N1-like wave changed over the course of the experiment, 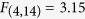, 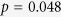, 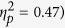, and hemisphere (reflecting that the N1-like wave was right-lateralized, 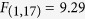, 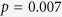, 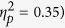. Moreover, the MANOVA indicated an interaction between block and hemisphere (reflecting that the lateralization of the N1-like wave was stronger at the beginning than at the end of the experiment, 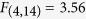, 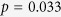, 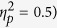. Despite a missing interaction between factors item position and block (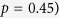, we carried out additional MANOVAs, separately for the first and second timbre (with factors block and hemisphere), justified by the large amplitude difference between the first and second triplet items (reflected in the large effect size of the main effect of item position). For the first item, the MANOVA indicated main effects of block 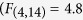, 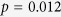 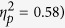 and hemisphere 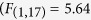, 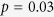, 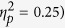. The amplitude increase of the N1-like wave (reflected in the main effect of block) followed a clear linear trend 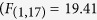, 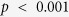, 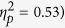. For the second item, neither an effect of block 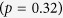, nor an interaction between block and hemisphere was indicated 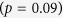.

A MANOVA for the P1 effect with factors item position (first and second triplet item), block (1 to 5), and hemisphere (left and right ROI) for the time window from 70 to 130 ms after the onset of first and second triplet items did not indicate an effect of item position 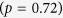, nor any interaction involving item position.

### Behavioral Effects of Ending Probabilities

**Figure 4:**
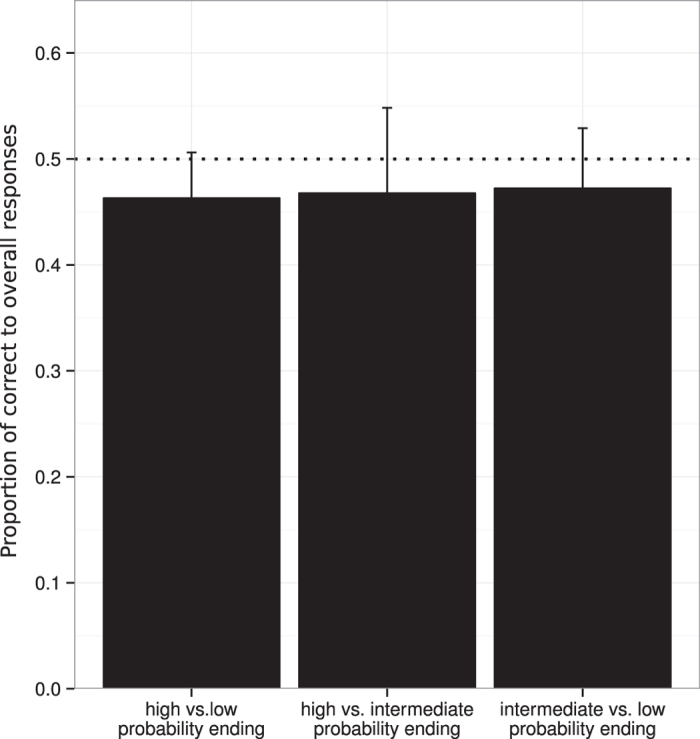
Results of the behavioral correctness (goodness-of-fit) ratings, separated by trial type. Error bars indicate 95% confidence intervals, the dotted line indicates chance level (0.5).

In the goodness-of-fit ratings, participants’ preference for triplet endings (i.e., the correctness of their choices) was not systematically influenced by probability. The proportion of correct responses was not significantly different from chance—neither overall, nor when analyzing trial types separately (for details see Table 3 and Fig. 4). Accordingly, an ANOVA revealed no effect of trial type on preference (correctness) judgments. There was no effect of repetition, nor an interaction between trial type and repetition.

For trials with high vs. low triplets there was a significant correlation between correct responses and confidence ratings 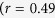, 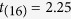, 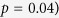. That is, in trials with high vs. low triplets subjects with higher numbers of correct responses also had higher confidence in their ratings. For the other trial types no such correlation was observed (see Table 3).

## Discussion

We used a statistical learning paradigm to compare the electroencephalographic and behavioral responses to acoustical events with high, intermediate, and low transitional probability. Probabilities were determined by how frequently a stimulus succeeded the preceding context of two stimuli. Our stimuli had no inherent hierarchical organization and were unknown to the participants. Thus, we tested whether participants would track statistical properties, in particular transitional probabilities throughout the experiment. We expected participants to successfully acquire such information and form a predictive model of the stimulus environment. Because processing stimuli based on a predictive model should facilitate processing, we also hypothesized that a processing advantage for more probable stimuli, and a relatively higher load for less probable stimuli, would be reflected in the electrical brain response to the final items (i.e., the endings) of the stimulus triplets. Correspondingly we found that items with lower probability elicited a stronger electrical brain response than items with high probability. This brain response had negative polarity, was distributed bilaterally over anterior scalp regions, had an onset at ~100 ms, and was maximal between ~130 to 220 ms after the onset of the final item. This response to less probable stimuli is consistent with results of previous MEG studies on statistical learning^29^,^30^,^31^,^32^, showing differences in magnetic brain responses between more predictable and less predictable items in a similar latency range (i.e., between ~100 to 220 ms). One of these studies also reported an earlier effect at ~50 ms^32^, but neither the other three MEG studies^29^,^31^,^32^, nor our ERP results showed such an early effect.

The effect observed in our study is reminiscent of a family of perisylvian negativities that have been associated with the processing of irregularities in the auditory domain^55^, namely the physical Mismatch Negativity (MMN), abstract-feature MMN, Early Left Anterior Negativity (ELAN), syntactic MMN, Left Anterior Negativity (LAN), and Early Right Anterior Negativity (ERAN). As described in the Introduction, the physical MMN and the abstract-feature MMN are elicited when stimuli deviate from regularities represented in the auditory sensory memory^56^. Thus, with regard to physical and abstract-feature MMN, such regularities can be established on-line, on a moment-to-moment basis through invariance of stimulus features or more complex and abstract interstimulus relations (for reviews see refs 35,36,44, for special cases see refs 39,40). However, in our experiment the irregularity of an event was not of an acoustical nature. Instead, representations of regularity had to be established through an extended period of listening and (implicit) learning of information beyond auditory sensory memory capabilities. This suggests an analogy between the negativity observed in the present study on the one hand, and the ERAN as well as the ELAN, the syntactic MMN and the LAN on the other, which all reflect the processing of syntactic irregularities represented in a long-term memory format. While ELAN, syntactic MMN, and LAN are associated with syntax processing in language^41^,^42^,^43^, the ERAN is associated with music-syntactic processing (e.g., relating the function of a chord to its harmonic context)^44^. Thus, stimuli used in experiments investigating ELAN, music-syntactic MMN, LAN, and ERAN also engage syntactic processing (such as morpho-syntactic processing) and possibly even hierarchical processing (due to the strong hierarchical organization of music and language)^47^,^48^,^49^. This makes it difficult to determine to which degree these ERP components reflect the pure processing of local dependencies (comparable to the processing of the statistical properties of stimuli in statistical learning paradigms), or also other cognitive processes. The negativity observed in the present study is purely due to the processing of local dependencies, i.e., purely due to the statistical properties of sounds, without any necessary contributions from hierarchical processing. Therefore, we suggest the term “statistical MMN” as a label for this mismatch response. However, this label comes with the note of caution that the MMN label is traditionally used for an effect elicited by irregularities that can be recognized on a moment-to-moment basis and stored in sensory memory (i.e., physical and abstract-feature MMN). It is likely that the sMMN does not only reflect higher processing load (e.g. due to the higher information content of unpredicted events), but also error signals (as, e.g., investigated within the framework of predictive coding)^21^,^22^,^57^. This issue remains to be specified.

Regarding the learning of the statistical properties of sounds across the exposition phase, ERPs elicited by the triplet root showed larger N1-like responses to the first than the second triplet item (cf. the dashed rectangles in Fig. 2C). Because the first and second triplet items were acoustically on average identical (see Fig. 1A), this observation indicates that participants had a cognitive representation of the triplet structure of the stimulus stream. Our data are consistent with data from adults (showing that ERP responses in the 100–200 ms range are more negative for the first than the second item of triplets, particularly in good learners)^23^, and newborns^28^. In addition, the N1-like response to the first (but not to the second) triplet item increased systematically across blocks. This increase probably reflects the development of word segmentation across blocks. Again, these observations are consistent with data showing enhanced N1 (and N400) ERP components in response to word onsets compared to random syllables (reviewed in ref. 26). With regard to effects of the learning of the statistical properties of the triplet endings across the experiment, we we found a trend for an interaction between block and probability (see also Fig. 3). This suggests that participants established representations of the statistical structure of the stream over the course of the exposition phase. Our observations of effects of statistical online learning are consistent with computational models of experimental data within the framework of implicit, or incidental learning^11^,^58^,^59^.

Incidental learning typically results in an intuition for the stimulus domain^14^. Therefore we hypothesized that the acquired knowledge about stimulus probability would invoke a gut feeling, strong enough to influence preference in a goodness-of-fit rating. This has been the case in previous studies^11^,^60^. However, our participants did not show such an effect. Similar dissociations between electrophysiological and behavioral results were also found in other studies^25^,^30^,^40^,^61^. In our experiment, participants learned incidentally, without instructions to do so, and while watching a movie. Although behavioral effects have been shown under similar conditions^62^, others found that inattention compromises statistical learning^63^. In our case, the participants’ knowledge might just not have reached the necessary stability to be accessible to behavioural measures. Also, our stimuli were artificial sounds that differed only subtly in timbre. Other studies show that memorizing and discriminating timbres is a difficult task^64^, and a previous study found larger amplitudes of effects elicited by low-probability items in response to pitches than to timbres (phonemes)^30^. Therefore, for future studies on statistical learning with auditory stimuli the use of pitch information, rather than timbre, seems advisable.

## Conclusions

Our study takes the statistical learning paradigm one step further: we constructed a new experimental setup to explore different degrees of intra-triplet probabilities within a continuous stream of triplets. We found an early negativity, referred to as “statistical MMN” (sMMN), that reflected probabilistic properties of sounds which were acquired through statistical learning under incidental learning conditions. Interestingly, the amplitude of the sMMN varied as a function of the frequency of occurrence (and thus the predictability) of the stimuli. Hence, our findings show that the sMMN reflects the detection of the degree of transition probability within the sequence. More specifically, our results indicate that representations of statistical regularities are reflected in a proportional neurophysiological response, the sMMN. Our findings are particularly interesting because, while being exposed to the stimulus, the participants were concurrently occupied with watching a movie, and therefore likely applied only a minimal amount of attention. Nevertheless, the brain responses of participants revealed that they had established representations of complex interstimulus relations, even *prior* to the participants being consciously aware that they had learned something, and even prior to clear behavioral evidence for learning, similar to a previous MEG study on statistical learning^32^, and similar to a study on abstract-feature contingencies^39^. Thus, our ERP results shed new light on the brain’s mechanisms involved in the acquisition and processing of statistical structures, as reflected in a statistical MMN.

## Additional Information

**How to cite this article**: Koelsch, S. *et al*. Under the hood of statistical learning: A statistical MMN reflects the magnitude of transitional probabilities in auditory sequences. *Sci. Rep.* **6**, 19741; doi: 10.1038/srep19741 (2016).

## Figures and Tables

**Table 1:** A: Means and standard deviations of the amplitudes in the time window used for statistical analysis (130–220 ms) after the onset of the triplet ending (i.e., after the onset of the third triplet item), separately for the three different ending probabilities (low, intermediate, high).

		**ROI mean**		**left ROI**		**right ROI**	
		***M***	***SD***	***M***	***SD***	***M***	***SD***
A	low	2.15	1.19	2.12	1.19	2.18	1.20
	intermediate	2.26	1.31	2.26	1.30	2.27	1.32
	high	2.64	1.35	2.62	1.35	2.66	1.35
B	block 1	0.18	0.55	0.27	0.55	0.10	0.58
	block 2	0.02	0.52	0.04	0.54	0.00	0.52
	block 3	0.03	0.71	0.06	0.69	0.01	0.75
	block 4	−0.18	0.44	−0.12	0.48	−0.23	0.42
	block 5	−0.30	0.57	−0.24	0.58	−0.36	0.58

B: Means and standard deviations of the amplitudes in the time window used for statistical analysis (170–230 ms) after the onset of the first triplet item, separately for the five blocks.

Note: Values in *μV*.

**Table 2:** Results of the evaluation of differences in mean amplitudes, comparing responses to triplet endings with different probabilities employing user-defined contrasts within the MANOVA.

	**ROI mean**				**left ROI**				**right ROI**			
	**diff.**	***F***_**(1,17)**_	***p***	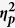	**diff.**	***F***_**(1,17)**_	***p***	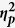	**diff.**	***F***_**(1,17)**_	***p***	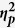
low < interm.	0.09	0.43	0.522	0.03	0.12	0.63	0.440	0.04	0.07	0.22	0.642	0.17
interm. < high	0.38	27.06	<0.001	0.61	0.37	21.57	<0.001	0.56	0.40	30.66	<0.001	0.64
low < high	0.48	10.27	0.005	0.38	0.49	8.90	0.008	0.34	0.46	11.45	0.004	0.40

Note: diff. = amplitude difference between the two conditions (in *μV*), interm. = intermediate.

**Table 3:** Results of the correctness (goodness-of-fit) and confidence ratings obtained after the exposition phase.

	**Proportion of “high-preferred” to overall responses**				**Confidence rating (on a scale from 1 to 6)**				
	***M***	***SD***	**difference from chance**		***M***	***SD***	**correlation with preference**		
			***t***_**(17)**_	***p***			***r***	***t***_**(16)**_	***p***
low vs. interm.	0.47	0.11	−1.03	0.32	3.64	0.64	0.12	0.47	0.65
interm. vs. high	0.47	0.16	−0.85	0.41	3.68	0.80	−0.11	−0.45	0.66
low vs. high	0.46	0.09	−1.81	0.09	3.74	0.62	0.49	2.25	0.04
overall	0.47	0.08	−1.76	0.10	3.69	0.67	0.20	0.83	0.42

Note: intem. = intermediate.
